# Kermit: linkage map guided long read assembly

**DOI:** 10.1186/s13015-019-0143-x

**Published:** 2019-03-20

**Authors:** Riku Walve, Pasi Rastas, Leena Salmela

**Affiliations:** 1 Department of Computer Science, Helsinki Institute for Information Technology HIIT, University of Helsinki, Helsinki, Finland; 2 Institute of Biotechnology, University of Helsinki, Helsinki, Finland

**Keywords:** Genome assembly, Linkage maps, Coloured overlap graph

## Abstract

**Background:**

With long reads getting even longer and cheaper, large scale sequencing projects can be accomplished without short reads at an affordable cost. Due to the high error rates and less mature tools, de novo assembly of long reads is still challenging and often results in a large collection of contigs. Dense linkage maps are collections of markers whose location on the genome is approximately known. Therefore they provide long range information that has the potential to greatly aid in de novo assembly. Previously linkage maps have been used to detect misassemblies and to manually order contigs. However, no fully automated tools exist to incorporate linkage maps in assembly but instead large amounts of manual labour is needed to order the contigs into chromosomes.

**Results:**

We formulate the genome assembly problem in the presence of linkage maps and present the first method for guided genome assembly using linkage maps. Our method is based on an additional cleaning step added to the assembly. We show that it can simplify the underlying assembly graph, resulting in more contiguous assemblies and reducing the amount of misassemblies when compared to de novo assembly.

**Conclusions:**

We present the first method to integrate linkage maps directly into genome assembly. With a modest increase in runtime, our method improves contiguity and correctness of genome assembly.

## Introduction

High-throughput, second generation, sequencing technologies made large scale de novo assemblies possible and commonplace. Their short read lengths however still pose a major problem to this day. Third generation, long read sequencing technologies, such as single-molecule real-time sequencing (SMRT) and Oxford Nanopore (ONT) are promising but their error rates have made assemblies difficult in practice. Therefore most long read assemblers include an error correction step to reduce the error rate.

Recently introduced Minimap–Miniasm workflow [1] has given new insight towards an error correction-free pipeline for long read assemblies. Minimap finds useful overlaps in long reads with high error rates and makes long read-only assembly projects practical and highly efficient. However finding the overlaps between reads with high error rates becomes impractical in very large data sets and splitting the reads into smaller sets is not possible without additional information on the reads.

Compared to de novo assembly, where the only available information for the assembly are the reads, *guided genome assembly* has additional data that gives information on the positions of the reads. Normally this additional data is a reference genome of a closely related species, see e.g. [2–4]. The reads can be aligned to the reference genome, which results in a linear ordering of the reads. This clearly makes it easier to then assemble the reads.

Directly guiding the assembly this way makes it hard to get an assembly that is higher quality than the reference. This becomes an issue when no high quality reference genome exists, or if the donor genome deviates too far from the reference genome. In this paper, we guide the assembly using linkage maps.

Linkage maps (also called genetic linkage maps or genetic maps) [5] are a useful technique to orient and place contigs within a chromosome and to detect misassembled contigs. The linkage maps themselves consist of variable genetic markers, typically called relative to some draft assembly. The markers are derived from a set of variations, such as single-nucleotide variations (SNVs), found from a sequenced *cross*, a population of related individuals. SNVs that are close to each other in the genome are more likely to be inherited together than SNVs that are more distant from each other. Linkage maps can therefore be constructed by genotyping the individuals in the cross and examining the probabilities of SNVs being inherited together.

Recently genotyping technologies have advanced so that more variations on larger crosses can be detected which allows for construction of much denser linkage maps than before. This has spurred the development of computational tools for linkage map construction for such dense data sets [5]. Dense linkage maps have been constructed for several recently sequenced genomes, including e.g. *M. cinxia* [6], *H. erato* [7], *B. pendula* [8], *T. cacao* [9], *G. aculeatus* [10] and *T. urartu* [11]. With a dense enough linkage map, it is possible to find approximate genomic locations for long reads directly.

In this paper, we formulate the genome assembly problem in the presence of linkage maps. We propose the first method to directly use linkage maps in genome assembly. Our method disentangles the overlap graph by removing spurious edges based on the linkage map. Our experimental results show that the method is able to simplify the overlap graph and we further show that our method decreases the number of misassemblies and improves the N50 statistic as compared to de novo assembly without linkage maps.

This linkage map-guided genome assembly can be seen as a generalisation of reference-guided genome assembly. With a hypothetical linkage map of evenly spaced markers, the linkage map-guided assembly becomes essentially reference-guided assembly as each read can be placed on the genome unambiguously.

We have implemented our method in a tool called Kermit which is freely available at https://github.com/rikuu/kermit.

## Related work

Despite the emergence of long read sequencing technologies like PacBio and ONT and the development of long read assemblers [1, 12–14], auxiliary long range data is still needed to organise the resulting scaffolds or contigs to chromosomes for large eukaryotic genomes. Depending on the characteristics of the species of interest such data may include optical mapping data, Hi-C data, or linkage maps.

Linkage maps consist of a set of markers, typically SNVs, on a genome. The location of the markers with respect to each other is at least approximately known. Typically a linkage map successfully divides the markers into chromosomes and a partial order of the markers within each chromosome is known. Markers can be localised on contigs or scaffolds and the linkage map can be used to correct the scaffolds and to order them into chromosomes [6–8]. However, currently detecting misassembled scaffolds and ordering of scaffolds based on a linkage map is a mostly manual process as no fully automated tools exist. Because de novo assemblies often contain thousands of scaffolds, this becomes time-consuming and error prone [15]. Furthermore, all current tools use linkage maps as a post processing step after assembly, whereas our work integrates the linkage maps already in the assembly phase.

Chromonomer [16] attempts to correct and orient scaffolds based on a linkage map. It first finds a non-conflicting set of markers in the linkage map. It then assigns orientation to scaffolds containing more than one recombination point and splits scaffolds if they conflict with the linkage map. Finally, Chromonomer produces a visualisation of the linkage map and the scaffolds. Another tool that facilitates the visualisation of linkage maps and corresponding genome assemblies is ArkMAP [17]. However, ArkMAP focuses on visual exploration including cross species comparisons and as such does not support automatic ordering of scaffolds into chromosomes. Both of these methods require visual inspection and manual work to order the scaffolds. Furthermore, if the genome assembler has omitted connections between contigs from the assembly because of conflicting information, these connections are not available at the post processing step anymore even if the linkage map could be used to resolve the conflicts.

Similarly to linkage maps also optical mapping data can be used for improving genome assemblies. Also for optical mapping data the main focus has been to use the optical map in a post processing step after genome assembly. AGORA [18] is one of the few methods integrating optical map data with genome assembly. It is a de Bruijn graph based assembler that uses optical map data to eliminate alternative paths that are not consistent with the optical map. The method by Alipanahi et al. [19] for disentangling the de Bruijn graph using optical map data is more related to our work. They map the long reads to the genome wide optical map and use this mapping to produce a positional de Bruijn graph which resolves most ambiguities in the de Bruijn graph. In our work we similarly first get a preliminary ordering of long reads based on a linkage map and then use this ordering to disentangle the overlap graph.

## Definitions

### De novo assembly

De novo assembly, the problem of assembling a genome given only a set of reads, has historically been given solutions in two different categories. The de Bruijn graph-based (DBG) assembly algorithms, such as SPAdes [20], and Overlap-Layout-Consensus (OLC) algorithms, such as Canu [12] and Miniasm [1].

OLC-assemblers attempt to first find pair-wise overlaps between reads, i.e. find a pair of reads $(u, v)$, where a suffix of $u$ and a prefix of $v$ are similar. From these overlaps, a directed graph of the set of reads, called an *overlap graph*, is laid out by assigning edges between reads if an overlap is observed. Note that as the orientation of reads is not known during this stage, we have two nodes in the graph for every read to cover both possible orientations.

Here we use a broader definition of an overlap graph than is traditional. Firstly, we allow the overlaps to be approximate, as we are using error-dense long reads. Secondly, we only add edges between reads that have sufficiently long overlaps and thus the graph is not complete.

Ideally the graph would be simple enough to unambiguously spell a genome assembly by following the edges from one side of the graph to the other. This is rarely the case but as long as we assume that there is an assembly path contained in the graph, we can attempt to remove edges based on different rules to hopefully be left with a simple assembly path. This pruning process is also referred to as the layout step.

DBG-assemblers attempt to simplify the entire process by not looking for overlaps between the reads. Instead they take all possible $k-1$ length overlaps between substrings of length $k$ from the set of reads to construct a de Bruijn graph. The methods for finding the genome from the overlap graph in the OLC setup mostly apply here too.

Though simpler than the OLC assembly algorithms, the effectiveness of further splitting the reads diminishes the usefulness of using long reads for genome assembly. For this reason we will be looking at the OLC category of assembly algorithms, more specifically, we are looking at Miniasm [1], a highly efficient implementation of an OLC-assembler.

Unlike traditional OLC-assemblers, Miniasm only implements the overlap and layout steps. This reduces the base-level quality of the resulting assembly but does not greatly affect the large scale structure. It also does not use any overly sophisticated method for finding the genome in the overlap graph.

To choose an assembly path, Miniasm finds *unitigs* which are maximal non-branching paths in the overlap graph. Such paths are simple to find and intuitively give the maximal unambiguous and non-repetitive sequences that the overlap graph can spell without changing the graph. The problem of finding the unitigs from the overlap graph can be stated as follows:

#### **Definition 1**

*Unitig assembly problem.* Given a directed graph $G = (V, E)$, find all maximal paths $P = v_1 v_2 \cdots v_n$ such that

$$\begin{aligned} \forall v_i \in \{v_1,\ldots ,v_{n-1}\}, \deg ^+{v_i} = \deg ^-{v_{i+1}} = 1, \end{aligned}$$

where $\deg ^+{v}$ and $\deg ^-{v}$ are the outdegree and indegree of $v$ respectively.

Unitigs can be efficiently found by first looking for a vertex with either zero or more than one incoming edge and exactly one outgoing edge. Following the outgoing edges until we find a vertex with more than one incoming edge or zero or more than one outgoing edge constructs a path spelling a unitig.

### Guided assembly

Reference-guided genome assembly can be done by aligning the read set to a reference and partitioning the reads based on the alignments into smaller, similar sets of reads [2]. The small sets are then assembled into contigs and later the set of contigs are assembled into super-contigs.

For linkage map-guided assembly, we partition the reads based on the linkage map. A *linkage map* is a set of markers $M$ which are assigned to a set of bins $B$. Each marker $m \in M$ belongs to a single bin $b \in B$. The bins are further assigned to chromosomes and within each chromosome the order of the bins is given.

We assume now that each read has been assigned a set of bins based on the linkage map. Now this coarse-grained ordering tells if a subset of reads clearly belong before or after another subset of reads. We encode this ordering into the overlap graph by assigning each vertex a set of colours representing the bins, resulting in a *coloured overlap graph*.

A coloured overlap graph is thus a directed graph $G=(V,E)$ accompanied by a colouring $c: V \rightarrow {\mathcal {P}}({\mathbb {N}})$, where ${\mathcal {P}}({\mathbb {N}})$ is the power set of natural numbers. In the coloured overlap graph we define the *colour consistent indegree* $\deg _c^-$ of a vertex $v$ to be the number of in-neighbours of $v_i$ that have at least one colour that is the same as or adjacent to a colour of $v_i$, i.e.

$$\begin{aligned} \deg _c^- v_i = |\{v_j| (v_j, v_i) \in E \text { and } \exists c_j \in c(v_j), c_i \in c(v_i) \text { s.t. } |c_j-c_i| \le 1 \}|. \end{aligned}$$

Similarily we define the *colour consistent outdegree* $\deg _c^+$ as

$$\begin{aligned} \deg _c^+ v_i = |\{v_j| (v_i, v_j) \in E \text { and } \exists c_j \in c(v_j), c_i \in c(v_i) \text { s.t. } |c_j-c_i| \le 1 \}|. \end{aligned}$$

The problem of guided assembly can then be modelled as finding a *rainbow path* in the coloured overlap graph. A rainbow path is a path such that no two vertices have the same colour [21]. We use a modified variant of rainbow paths; we allow paths to reuse a colour in consecutive vertices and we require the colours of a path to be consecutive and increasing. I.e. colour $i$ must be followed by colour $i+1$ on the path. A more formal definition of this assembly problem can be stated as:

#### **Definition 2**

*Guided unitig assembly problem.* Given a directed graph $G = (V, E)$, and a colouring $c : V \rightarrow {\mathbb {N}}$, find all maximal paths $P = v_1 v_2 \cdots v_n$ such that

$$\begin{aligned} \forall v_i \in \{ v_1,\ldots ,v_{n-1} \}, \deg _c^+{v_i} = \deg _c^-{v_{i+1}} = 1, \end{aligned}$$

and

$$\begin{aligned} \forall c \in c(v_i), c \ge \max \left( c(v_{i-1}) \right) . \end{aligned}$$

We note that the above definition only recovers paths in one direction in the coloured overlap graph. However, because we add each read both in the forward and reverse orientation to the overlap graph, for each reverse complementary path there also exists a corresponding forward strand path in the graph.

Rather than modifying the layout step of the OLC-assembly pipeline, we implement a graph cleaning step, which removes edges that make unitigs non-rainbow path unitigs.

## Methods

### Overview of our method

The input to our method is a draft assembly, a linkage map, and long reads. The draft assembly can be generated from any read data from the same species. For example, it can be generated from the same long reads given also as input to our method or from the sequencing data used for calling the markers of the linkage map. The draft assembly can be a low quality assembly with many short contigs and even some assembly errors.

Constructing a linkage map involves localising the markers, which are typically single nucleotide variations, on the draft assembly. The markers are then further placed into bins based on the hereditary patterns seen in a cross-bred population of individuals. The bins are assigned to chromosomes and the order of the bins within each chromosome is known. Thus the markers give a partial order of the genome. The linkage map should be dense. It should contain sufficiently many markers so that most reads contain at least one marker location. Such dense linkage maps can be constructed for example by Lep-MAP3 [5]. We note that whereas the draft assembly contains only short range information spanning a single contig, the linkage map gives a global view of the genome spanning whole chromosomes. Thus the linkage map is superior to the draft assembly as a reference for guiding the assembly in our method.

**Fig. 1:**
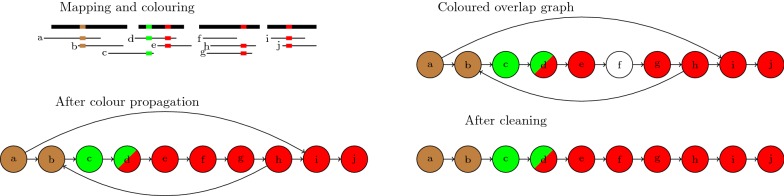
Overview of our method. First the reads are mapped to the draft assembly and assigned colours (top left). Each colour represents one bin in the linkage map. In this example we have three bins (brown, green and red) and the ordering of the bins is brown < green < red. All bins belong to the same chromosome. Miniasm is then used to construct the overlap graph which is augmented with the colours. Next vertex $f$ is coloured through the colour propagation process. Finally we remove the edges $(a, i)$ and $(h, b)$ because they have inconsistent colourings

Figure 1 shows an overview of our method. We first use Minimap2 [22] to map the long reads on the draft assembly to assign colours (i.e. bin numbers) to the long reads. Miniasm [1] is then used to build the overlap graph of the long reads and missing colours are propagated in the overlap graph. The overlap graph is then cleaned based on the colour assignments of the reads. Finally the unitigs are read from the cleaned overlap graph. The colouring, propagation, and cleaning steps are discussed in more details in the following subsections.

### Colouring

To colour the underlying overlap graph, we map all the reads against the draft assembly using Minimap2 [22] and store the longest mappings for each read. We extend each of the longest mappings into naive linear “alignments” by stretching the start and end positions to cover the entire read. As the insertion errors are the dominant error type [23], we limit the number of characters by which we extend the mappings (by default we use a limit of 250 bp).

All the extended mappings are then stored in a simple query structure, where each chromosome in the reference genome is split into equal length blocks. For each marker in the linkage map, we query the index for reads that overlap with the marker and assign every overlapping read the colour the marker in the linkage map belongs to.

As there can still be reads with no mapping to the reference, we attempt to colour them using the overlap graph. For each uncoloured vertex $u$ we find the set of coloured vertices $V_c(u)$ that are reachable from $u$ by paths that traverse only uncoloured vertices. The uncoloured vertex $u$ is then given all the colours that the vertices in $V_c(u)$ have, i.e. $c(u)=\bigcup _{v\in V_c(u)} c(v)$. The set $V_c(u)$ can be found by a breadth first search on the graph starting at vertex $u$.

If a coloured vertex is too far from the uncoloured vertex, we get an over-approximation of the colour for the vertex. To reduce this effect, we apply a limit to the depth of the search (by default 10). We can also get conflicting colours from the propagation. If there are missing colours between the propagated colours, we are skipping some part of the genome entirely. As we cannot use the colouring to usefully clean the graph, we simply remove the read from the graph entirely.

### Graph cleaning

To make sure any unitig path is a valid rainbow path, we remove any edges between vertices with inconsistent colourings from the overlap graph. If the vertices share a colour, the colourings are always consistent as they can be merged without affecting the connectivity of the colours in the graph.

Edges between vertices with different colours are inconsistent if there are no adjacent colours between the vertices. Such an edge could never be part of a rainbow path because the path would not have consecutive colours.

Edges with colourings further apart may still be considered correct, as the linkage maps may have noisy marker positions. Therefore we define an edge $(v_i,v_j)$ to be *inconsistently coloured* if there are no colours $c_i \in c(v_i)$ and $c_j \in c(v_j)$ such that the colours $c_i$ and $c_j$ would be within some distance $d$, i.e. $\mid c_i - c_j \mid \le d$. Our graph cleaning step removes all inconsistently coloured edges from the overlap graph.

The set of edges in the overlap graph can be divided into two disjoint subsets: the genomic edges which connect reads that are derived from overlapping positions in the genome and the additional spurious edges caused by reads that overlap although they are derived from distinct positions in the genome.

#### **Theorem 1**

*If all reads are correctly coloured and the overlap graph contains at least one genomic incoming and outgoing edge for each node that is not the endpoint of a chromosome, then removing all inconsistently coloured edges results in all unitigs being rainbow unitigs.*

#### *Proof*

When all inconsistently coloured edges have been removed from the set of spurious edges, the only remaining spurious edges are those that are not inconsistently coloured. These edges represent spurious overlaps between reads that have consistent colours.

Let $(u,v)$ be a spurious edge that is not inconsistently coloured and thus it may falsely connect two rainbow paths in the overlap graph. First we note that if both $u$ and $v$ are endpoints of chromosomes, then the edge $(u,v)$ must be inconsistently coloured because we assumed all reads are correctly coloured. Thus either $u$ or $v$ must not be an endpoint of a chromosome.

Without loss of generality let us assume that $u$ is not an endpoint of a chromosome. Because all nodes have an outgoing genomic edges in the overlap graph, there must be another edge $(u,v')$ in the graph. Thus $u$ is a branching node and a unitig cannot contain edge $(u,v)$. Therefore a unitig cannot contain any spurious edges. Because unitigs now consist of only genomic edges and all reads are correctly coloured, all unitigs must be rainbow unitigs. $\square$

## Results

We implemented our method in a tool called Kermit. Kermit uses Miniasm [1] to find the overlaps between the reads and to perform the layout step to find unitigs. In between these two steps Kermit colours the vertices of the overlap graph and cleans the graph by removing inconsistently coloured edges as explained in the previous section.

We compared Kermit to Miniasm [1] and Canu [12] which ranked the best in the average rankings of all assemblies in a recent survey on long read assembly by Jayakumar and Sakakibara [24]. For Kermit and Miniasm, both the overlap and the mapping steps were done using Minimap2 v2.12 [22], an improved implementation of Minimap. All results are given using default settings for the tools. We tried varying the minimum overlap parameter and the minimal coverage of overlaps in Miniasm but found that the default settings gave the best performance. All experiments were run on 32 GB RAM machines equipped with 8 cores.

### Data

We performed experiments both on simulated and real data. All data used in the experiments is summarised in Tables 1 and 2.

**Table 1:** Summary of read data used in the experiments

Data set	Reads	Total bases	Coverage	Accession
*S. cerevisiae* (simulated)	36,639	486,048,334	39.98	Simulated
*C. elegans* (simulated)	296,230	3,930,689,562	39.99	Simulated
*S. cerevisiae*	52,208	690,899,144	56.83	PacBio $\text {DevNet}$^a^
*C. elegans*	740,776	8,118,404,281	82.59	PacBio $\text {DevNet}$^b^
*H. erato*	10,818,653	27,094,241,328	60.89	SRR3476970 SRR4039325
*B. pendula*	1,898,360	19,032,363,776	49.71	ERR2003767 ERR2003768

^a^ https://github.com/PacificBiosciences/DevNet/wiki/Saccharomyces-cerevisiae-W303-Assembly-Contigs

^b^ https://github.com/PacificBiosciences/DevNet/wiki/C.-elegans-data-set

**Table 2:** Summary of linkage maps used in the experiments

Data set	Markers	Marker density	Bins	Bin density	References
*S. cerevisiae* (simulated)	100,009	0.008	19,283	0.002	Simulated
*C. elegans* (simulated)	750,004	0.008	162,601	0.002	Simulated
*H. erato*	2,781,314	0.007	145,863	0.002	Van Belleghem et al. [7] Generated by LepMap3 [5]
*B. pendula*	2,979,993	0.007	925,123	0.002	Salojärvi et al. [8]

First, we performed experiments using both simulated reads and a simulated linkage map for *S. cerevisiae* and *C. elegans*. We simulated reads using SimLoRD [23] to a coverage of 40x, sampling read lengths from a real PacBio data set with shortest (< 10,000 bp) reads filtered out.

To generate a simulated linkage map, markers were randomly placed on the reference and binned such that the bins are separated by at least 200 bp. The number of markers was chosen to give a marker density similar to real linkage maps. The size of bins was chosen such that the resulting densities of bins are similar to the real linkage maps as shown in Table 2. These experiments on simulated read and linkage map data allow us to evaluate the colouring and cleaning steps because the origin of each read is known.

To understand how our method performs on real data, we first ran experiments using real PacBio reads for *S. cerevisiae* and *C. elegans* but still using the simulated linkage map as we are not aware of dense enough real linkage maps for these species. Good reference genomes are available for these genomes so we could evaluate also the correctness of the resulting assemblies on these data sets.

Finally, to show that our method works on real linkage maps, we ran experiments on real PacBio reads and real linkage maps for *H. erato* and *B. pendula*. The genomes for these species are in draft stage so we could not reliably measure the correctness of these assemblies.

### Colouring

We first evaluated the results of the colouring and propagation steps on the simulated reads and linkage maps. Because the reads are simulated, we know for each read the position where it derives from. Therefore we can also deduce the correct colours for each read as follows. For every read there are two markers in the linkage map that form the upper and lower limit of acceptable colours the read can get. All the colours should be between the colour of last marker before the read and the first marker after the read.

We are mostly interested in the set of reads that are fully within the correct range, i.e. no colour given to the read is incorrect, and the set of reads that are fully outside the correct range.

As can be expected, Table 3 shows that a vast majority of the reads are completely inside their colour ranges. The small amount of reads outside the range are reads that have been mapped to an entirely wrong position of the draft assembly.

**Table 3:** Number of reads fully inside and outside their acceptable colour ranges

	Reads inside	Reads outside
*S. cerevisiae*	36,562 (99.79%)	31 (0.08%)
*C. elegans*	296,134 (99.97%)	3 (0.001%)

### Cleaning

To evaluate the effectiveness of removing colour crossing edges, we find the simulated positions of the two reads corresponding to each edge in the graph and check whether they overlap in the reference genome. We consider those overlapping reads to be genomic in the sense that using that single edge in a contig would spell the correct sequence.

Miniasm already implements a number of cleaning methods which are solely based on the topology of the overlap graph. We counted the number of genomic and spurious edges both with and without these cleaning steps to understand how each cleaning step affects the overlap graph.

Table 4 shows how Kermit is able to improve the percentages of genomic edges by removing spurious edges in the graph. Though the amounts of spurious edges removed is relatively small, any single wrong edge used in the assembly can cause a misassembly or break a unitig.

**Table 4:** Number of edges supported by the positions the reads were simulated from

	Graph	Genomic edges	Spurious edges
*S. cerevisiae*	Miniasm	76,538 (99.39%)	466 (0.60%)
	Kermit	76,518 (99.93%)	52 (0.07%)
	Miniasm cleaned	7146 (99.92%)	6 (0.08%)
	Kermit cleaned	7114 (100.0%)	0 (0.0%)
*C. elegans*	Miniasm	668,012 (99.80%)	1306 (0.19%)
	Kermit	667,970 (99.99%)	58 (0.003%)
	Miniasm cleaned	60,416 (99.95%)	28 (0.05%)
	Kermit cleaned	60,356 (99.997%)	2 (0.003%)

Graphs marked cleaned are also using the graph cleaning steps that are already implemented in Miniasm

### Assembly

Lastly, we compare the actual assemblies produced by our tool to those produced by Miniasm and Canu. To get a better picture of the assemblies, we also applied a consensus tool, Racon v1.3.1 [25], on the *S. cerevisiae* and *C. elegans* assemblies produced by our tool and Miniasm. On the *H. erato* and *B. pendula* assemblies Racon was very slow so we did not run it on those assemblies. The assembly statistics were generated with QUAST v5.0 [26].

Table 5 shows how the assembly statistics are improved using Kermit on the simulated *S. cerevisiae* and *C. elegans* reads and simulated linkage maps. The number of contigs is reduced and the NGA50 statistic is increased indicating a more contiguous assembly. Also the number of misassemblies is either reduced or stays the same.

**Table 5:** Assembly statistics for simulated *S. cerevisiae* and *C. elegans* reads and simulated linkage maps

Assembly	*S. cerevisiae*		*C. elegans*	
	Miniasm	Kermit	Miniasm	Kermit
# of contigs	26	22	291	261
Total length	11,831,837 (97.32%)	11,728,421 (96.47%)	102,040,817 (103.81%)	101,632,493 (103.40%)
N50	605,399	640,779	2,337,914	2,293,633
NGA50	565,122	585,849	2,070,983	2,337,914
Misassemblies	2	1	7	7

Next, we evaluated Kermit on the real read data and simulated linkage map of *S. cerevisiae* and *C. elegans*. Table 6 shows that when compared to Miniasm the number of contigs and the number of misassemblies is reduced, whereas the NGA50 statistic is increased on the *S. cerevisiae* data. On the *C. elegans* data Kermit is more conservative. It reduces the number of misassemblies but the NGA50 statistic is slightly decreased and the number of contigs is slightly increased. Canu produces assemblies with comparable NGA50 values but it produces more misassemblies than Miniasm or Kermit.

**Table 6:** Assembly statistics for real *S. cerevisiae* and *C. elegans* reads and simulated linkage maps

Assembly	*S. cerevisiae*			*C. elegans*		
	Miniasm	Kermit	Canu	Miniasm	Kermit	Canu
# of contigs	31	29	34	177	188	159
Total length	12,118,143 (99.68%)	11,997,376 (98.69%)	12,426,814 (102.22%)	109,318,925 (111.22%)	104,545,368 (106.36%)	108,154,535 (110.03%)
N50	732,688	763,111	739,529	2,270,602	1,928,805	3,202,659
NGA50	345,801	376,210	375,952	272,995	271,763	271,783
Misassemblies	65	60	82	1837	1651	2019

Finally, we ran experiments on real read data and real linkage maps (*H. erato* and *B. pendula*). For these species only draft assemblies are available and thus we could not validate the produced assemblies and compute the number of misassemblies or the NGA50 statistics. Table 7 shows that for both data sets Kermit reduces the number of contigs and increases N50 as compared to Miniasm. Both of these measures indicate a more contiguous assembly. Canu produces more fragmented assemblies than Miniasm or Kermit on both of these data sets.

**Table 7:** Assembly statistics for real *H. erato* and *B. pendula* reads and real linkage maps

Assembly	*H. erato*			*B. pendula*		
	Miniasm	Kermit	Canu	Miniasm	Kermit	Canu
# of contigs	7444	6091	100,615	2201	1587	14,189
Total length	327,725,353 (86.24%)	280,881,758 (73.92%)	691,789,561 (180.69%)	473,300,369 (107.57%)	425,356,395 (96.67%)	387,624,902 (87.11%)
N50	58,892	60,356	12,592	435,830	539,400	45,255

For the *B. pendula* set, our machine would run out of memory when constructing the graph with Miniasm and Kermit. This was alleviated by using the option to remove clearly contained reads.

We also note that all of the assemblers struggle on the *H. erato* data. We believe this is largely due to the fact that we needed to pool PacBio reads from two experiments using two different individuals to have enough reads for assembly. This introduces heterogeneity to the data and makes assembly challenging.

We also investigated the effect of the distance parameter for determining colouring inconsistency using the real *B. pendula* dataset. In a real linkage map, the boundaries of colours can be noisy and thus markers with nearby colours could be in the wrong order. We looked at assemblies using four different values for the distance ($d=0,1,2,3$) and a setting where all chromosomes are given a single colour and $d=0$. This latter represents the largest distance we could possible allow, as only edges that connect non-contiguous regions of the genome are removed. The results are summarised in Table 8.

**Table 8:** Assembly statistics for *B. pendula* dataset with different colour distance parameters

Assembly	Unicoloured	$d=0$	$d=1$	$d=2$	$d=3$
# of contigs	1583	1594	1587	1585	1583
Total length	426,546,974	425,333,715	425,356,395	425,531,261	425,708,460
N50	537,429	539,400	539,400	538,989	538,770

The unicoloured column shows results for colouring each chromosome with a single colour

We see that the distance parameter does not have a large effect on the assembly contiguity which indicates that our method is robust with respect to the mixing of nearby colours in real linkage maps. Furthermore, when using a single colour for each chromosome the N50 value is only slightly decreased as compared to the more fine grained schemes. As misassemblies connect random parts of the genome, most of them will span two chromosomes. Therefore it is not surprising that using a single colour for each chromosome results in almost equally good contiguity as more fine grained schemes.

### Running time

We recorded the wall clock time for all experiments. Table 9 shows that Kermit needs at most 5% more time than Miniasm on all data sets except *H. erato* on which it needs 13% more time. In some cases the total running time is even reduced.

**Table 9:** Wall clock times for all steps taken by the tools

	Tool	Overlap	Map	Colour	Layout	Consensus	Total
*S. cerevisiae* (simulated)	Miniasm	52 s	–	–	6 s	4 min 52 s	5 min 50 s
	Kermit	52 s	6 s	0 s	6 s	3 min 29 s	4 min 38 s
*C. elegans* (simulated)	Miniasm	9 min 55 s	–	–	1 min 58 s	28 min 19 s	40 min 17 s
	Kermit	9 min 55 s	2 min 17 s	5 s	55 s	28 min 57 s	42 min 9 s
*S. cerevisiae*	Miniasm	1 min 9	–	–	8 s	2 min 45 s	4 min 2 s
	Kermit	1 min 09 s	10 s	1 s	8 s	2 min 44 s	4 min 12 s
	Canu	–	–	–	–	–	2 h 12 min
*C. elegans*	Miniasm	16 min 54 s	–	–	4 min	53 min 28 s	1 h 14 min
	Kermit	16 min 54 s	4 min 8 s	11 s	2 min 29 s	50 min 29 s	1 h 14 min
	Canu	–	–	–	–	–	12 h 31 min
*H. erato*	Miniasm	8 h 40 min	–	–	1 h 30 min	–	10 h 10 min
	Kermit	8 h 40 min	9 min	2 min	2 h 42 min	–	11 h 32 min
	Canu	–	–	–	–	–	7 days
*B. pendula*	Miniasm	3 h 24 min	–	–	1 h 32 min	–	4 h 57 min
	Kermit	3 h 24 min	9 min	17 s	1 h 37 min	–	5 h 11 min
	Canu	–	–	–	–	–	6 days

The consensus phase was very slow on the big genomes of *H. erato* and *B. pendula* so it was not run on those data sets

Compared to Canu, both Miniasm and Kermit are significantly faster. This is likely due to Canu having a read error correction step. However, we did not run Racon on the *H. erato* and *B. pendula* assemblies of Miniasm and Kermit, whereas Canu pipeline includes error correction and consensus steps which contribute to its higher running time. Additionally, we see that the consensus step using Racon easily dominates the Miniasm and Kermit pipelines in terms of time to complete.

## Conclusions

We defined guided assembly with linkage maps by extending the unitig assembly model. Our method, Kermit, is implemented as a graph cleaning step and the contigs are generated with a simple unitig algorithm. As such the graph cleaning step could be used as a preprocessing step of a more complicated traversal algorithm for retrieving the contigs. Colouring the reads also leads naturally into non-overlapping bins of reads, that can be assembled independently. This allows massive parallelism in the assembly and could make more sophisticated assembly algorithms practical.

When defined as an independent graph cleaning step, our method guiding the assembly could be applied not only to other OLC-assemblers, but also to DBG-assemblers. In this case, the colours would be assigned to reads and the $k$-mers would get all colours present in reads where they derive from.

The colouring can also be used for ordering the assembled unitigs. While the current implementation only reports the colours for each unitig, these unitig colourings could be used to also connect the unitigs into more complete contigs.

Our experiments show that Kermit successfully removes spurious edges from the overlap graph. Furthermore we showed that with only a modest increase in runtime Kermit improves the contiguity and correctness of assembly as compared to current de novo long read assemblers.
